# The Board of Draft

**Published:** 1863-11

**Authors:** 


### THE BOARD OF DRAFT.

The Board of Draft has concluded its labors in this district, so far as hearing applications for exemptions, receiving substitutes, &c., was concerned. The following, furnished by Provost Marshall Scroggs, shows the result:

| | | | |
|---|---|---|---|
| Physical disability | 1,187 | Passed for duty and furnished substitutes | 443 |
| Mental disability | 19 | Paid $300 | 217 |
| Only son of widow | 85 | Non-residents | 101 |
| Only of aged or infirm parents | 102 | Alienage | 393 |
| One of two sons drafted and then elected | 31 | In service 3d of March | 28 |
| One of two sons elected after draft | 28 | Convicted of felony | 1 |
| Brother of children dependent on him | 1 | Died before draft | 1 |
| Father of motherless children | 20 | " after draft | 1 |
| To of same household in service | 12 | Passed for duty and not finally reported | 60 |
| Over 35 years of age and married | 297 | | |
| Under 20 | 49 | Total | 3,120 |

The whole number drafted in this county was 3,808, of which, as appears from the above table, 443 have furnished substitutes; 217 paid the commutation fee, and 60 passed for duty, and are now on furlough—making a total of 270, as the proceeds of the draft. Six hundred and eighty-eight drafted men have failed to report to the Board, and are now classed as deserters; 1,894 have been exempted on legal grounds, and 1,206 on account of physical and mental disability. The whole expenses of the Board of the Board of Enrollment have been $12,000; and the expense per man realized from the draft, $16 66.

